# Efficacy and Safety of Nifedipine Compared to Intravenous Hydralazine for Severe Hypertensive Disorders in Pregnancy: A Systematic Review and Meta-Analysis of Randmomized Controlled Trials

**DOI:** 10.3390/medsci13030091

**Published:** 2025-07-13

**Authors:** Vaisnavy Govindasamy, Mohammed Amer Kamel, Gabriele Volucke, Aashir Javed, Upayan Palchaudhuri, Sayed Irfan Kazi, Ahmad Albanna, Mays Akileh, Rohit Mukherjee, Rabia Nusrat, Tayyaba Qaiser, Eman Ibrahim Elzain Hassan, Muhammad Muneeb Azhar, Tallal Mushtaq Hashmi, Mushood Ahmed, Ali Hasan, Raheel Ahmed

**Affiliations:** 1 Department of Medicine, James Cook University Hospital, Middlesbrough TS4 3BW, UK; vaisnavygovin@gmail.com; 2 Department of Medicine, Al-Quds University, East Jerusalem 20002, Palestine; mohammed.kamel@students.alquds.edu; 3 Department of Cardiology, University Hospitals Plymouth NHS Trust, Plymouth PL6 8DH, UK; volucke@gmail.com; 4 Department of Medicine, University Hospital of North Durham, Durham DH1 5TW, UK; javedaashir522@gmail.com; 5 South Tyneside and Sunderland NHS Foundation Trust, Tyne & Wear SR4 7TP, UK; upayan.palchaudhuri1@nhs.net; 6 Department of Cardiology, Royal Berkshire Hospital, Reading RG1 5AN, UK; irfan.kazi@gmail.com; 7 Department of Medicine, The University of Jordan, Amman 11942, Jordan; dr.albanna10@gmail.com (A.A.); maysakileh@yahoo.com (M.A.); 8 Department of Medicine, King Hussein Cancer Center, Amman 11941, Jordan; 9 Department of Medicine, Northumbria Specialist Emergency Care Hospital, Newcastle upon Tyne NE23 6NZ, UK; rohitmukherjee808@gmail.com; 10 Department of Obstetrics and Gynaecology, Southend University Hospital, NHS Foundation Trust, Southend-on-Sea NW1 3AX, UK; rabianusrat@yahoo.com; 11 Department of Medicine, Jinnah Sindh Medical University, Karachi 75510, Pakistan; tayyaba.qaiser5@gmail.com; 12 Department of Cardiology, Sheikh Shakhbojt Medical City, Abu Dhabi 11001, United Arab Emirates; dr.eman.i.elzain@gmail.com; 13 Sheikh Zayed Medical College, Rahim Yar Khan 64200, Pakistan; muneebazhar143@gmail.com; 14 Department of Medicine, Rawalpindi Medical University, Rawalpindi 46000, Pakistan; tallalhashmi12@gmail.com (T.M.H.); mushood07@gmail.com (M.A.); 15 Department of Cardiology, Imperial College London, London SW7 2AZ, UK; ali.hasan21@imperial.ac.uk

**Keywords:** gestational hypertension, preeclampsia, hypertension

## Abstract

**Background:** Severe maternal hypertension is linked to adverse perinatal outcomes. Both nifedipine and hydralazine are commonly used antihypertensive agents in this setting. **Methods:** A comprehensive literature search was conducted in PubMed, Cochrane Library, and EMBASE from inception to April 2024 to identify randomized controlled trials comparing oral or sublingual nifedipine with intravenous hydralazine for the management of severe hypertension, with or without preeclampsia/eclampsia. A random-effects meta-analysis was performed using RevMan. **Results:** Seven randomized controlled trials were included. The pooled analysis demonstrated no significant difference between the two agents regarding time to achieve optimal blood pressure control (MD = −1.08 min, 95% CI = −6.66 to 4.49), caesarean delivery (OR = 0.62, 95% CI = 0.38 to 1.03), neonatal birth weight (MD = 57.65 g, 95% CI = −209.09 to −324.40), NICU admissions (OR = 0.90, 95% CI = 0.41 to 1.98), and 5-min APGAR scores (MD = 0.1, 95% CI = −0.20 to 0.39). However, patients receiving nifedipine had significantly lower odds of experiencing medication-related adverse events (OR = 0.62, 95% CI = 0.40 to 0.97). **Conclusions:** Nifedipine and intravenous hydralazine showed comparable efficacy in achieving optimal blood pressure control and similar maternal and neonatal outcomes. However, nifedipine was associated with significantly fewer maternal adverse effects, indicating superior tolerability.

## 1. Introduction

Hypertensive disorders of pregnancy, especially severe pre-eclampsia and eclampsia, are among the leading causes of maternal and perinatal mortality worldwide [1]. An estimated 14% of maternal deaths globally (over 60,000 deaths each year) are attributable to these conditions [1,2]. Severe hypertension in pregnancy (typically defined as blood pressure ≥ 160/110 mmHg) precipitates acute end-organ damage, most ominously intracerebral hemorrhage, and is a true obstetric emergency [2]. Without prompt blood pressure control, the risk of maternal stroke escalates significantly, contributing to the high morbidity and mortality associated with these disorders [3].

Early treatment of severe pregnancy hypertension can significantly reduce maternal intracranial hemorrhage. Common antihypertensives used as first-line therapy include intravenous hydralazine, intravenous labetalol, and immediate-release oral nifedipine. However, targeted antihypertensive treatment is essential for maternal safety [2,3]. Hydralazine, a direct vasodilator, requires intravenous access and careful titration, while nifedipine is a quick-acting, easy-to-administer alternative. Both drugs have a rapid onset of action and short dosing intervals, making them commonly used as first-line antihypertensives in low- and middle-income countries [4].

Both hydralazine and nifedipine are accepted initial therapies for acute blood pressure control in severe pre-eclampsia, with the choice often based on clinician preference and clinical context. Recent evidence suggests that both drugs are highly effective in preventing maternal hypertensive crises, with generally comparable outcomes. A randomized clinical trial found that oral nifedipine achieved blood pressure targets as successfully as intravenous hydralazine, with no significant differences in time to control, maternal adverse events, or perinatal outcomes [2]. While previous meta-analyses [4,5,6] have compared the efficacy of several antihypertensive agents at treating maternal hypertension and lowering blood pressure to target levels, meta-analytic evidence directly comparing the efficacy of nifedipine with IV hydralazine across different maternal and fetal outcome measures is limited. Therefore, we aim to provide a direct comparison of the comparative efficacy and relative safety of nifedipine and intravenous hydralazine in pregnancy.

## 2. Methods

This meta-analysis was conducted following the Preferred Reporting Items for Systematic Reviews and Meta-analysis (PRISMA) recommendations [7]. The protocol has been registered on PROSPERO (ID: CRD420251065893) to ensure transparency and methodological rigor. This meta-analysis did not involve direct patient recruitment or data collection by the authors; therefore, formal ethical approval was not required.

### 2.1. Data Sources and Searches

We searched the following databases from inception till April 2025: Cochrane Central Register, PubMed, and Embase. A comprehensive literature search was conducted using a combination of Medical Subject Headings (MeSH) and free-text terms such as “nifedipine,” “hydralazine,” “severe hypertension,” and “randomized controlled trial” (Table S1). Boolean operators (AND, OR) were applied to optimize sensitivity and specificity in identifying relevant studies.

### 2.2. Eligibility Criteria

The inclusion criteria were as follows: population: pregnant females diagnosed with severe hypertension (defined as a systolic blood pressure ≥ 160 mmHg or diastolic blood pressure ≥ 110 mmHg) with or without pre-eclampsia or eclampsia; intervention: oral/sublingual nifedipine; comparator: intravenous hydralazine; outcome: Reports at least one relevant clinical outcome; study design: randomized controlled trials. Studies were excluded if they employed any design other than randomized controlled trials, including quasi-randomized trials, case reports, case series, and observational studies.

### 2.3. Selection Process

All records retrieved from the initial search were imported into Rayyan for deduplication and screening. Following removal of duplicates, two reviewers independently screened the titles and abstracts. Full-text articles of potentially eligible studies were then reviewed in detail by the same reviewers. Any disagreements were resolved through discussion with a third reviewer.

### 2.4. Data Extraction

Data on study characteristics, including authors and study locations, intervention, sample size, age, gestational age, and dosing strategies, were extracted. Data was organized into a pre-piloted Excel sheet. The maternal outcomes included time required for optimal blood pressure control, caesarean delivery, and medication adverse events. Neonatal outcomes included birth weight, NICU admissions, and 5-min APGAR score.

### 2.5. Risk of Bias Assessment

The quality assessment of included trials was conducted using the Cochrane Risk of Bias 2.0 (RoB 2.0) tool by two independent reviewers [8]. Any discrepancies were resolved through consultation with a third reviewer.

### 2.6. Data Analysis

The Statistical analyses were performed using Review Manager (RevMan), the official software developed by the Cochrane Collaboration. Odds ratios (ORs) with 95% confidence intervals (CIs) were calculated for binary outcomes, while continuous outcomes were pooled using weighted mean differences (WMDs) with corresponding 95% CIs. A random-effects meta-analysis was conducted to account for potential heterogeneity across the trials. The I^2^ statistic was used to assess the statistical heterogeneity among trials. Publication bias was not assessed using funnel plots or statistical tests due to the inclusion of fewer than ten studies. To evaluate the robustness of the findings and explore potential sources of heterogeneity, a leave-one-out sensitivity analysis was performed.

## 3. Results

### 3.1. Study Selection

**Figure 1:**
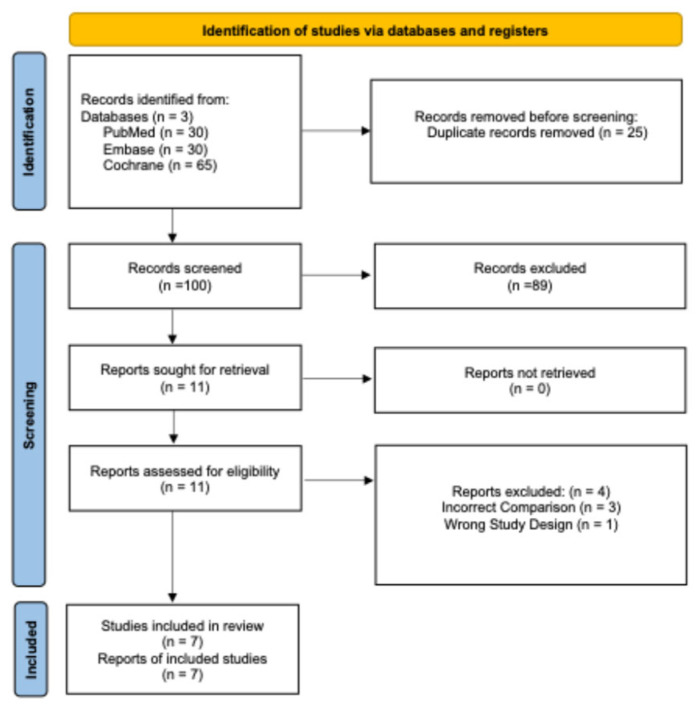
PRISMA flowchart depicting the screening and study selection process.

A total of 125 articles were initially retrieved through database searches. Following the removal of 25 duplicate entries, 100 records were subjected to title and abstract screening. Of these, 89 were excluded, resulting in 11 articles selected for full-text review. After a thorough assessment, 7 studies met the inclusion criteria and were incorporated into the final analysis. (Figure 1).

### 3.2. Study Characteristics

A total of seven randomized controlled trials [2,9,10,11,12,13,14] were included in the analysis. These studies were conducted between 1992 and 2020 and included a combined total of 727 participants receiving either oral/sublingual nifedipine or intravenous hydralazine for management. Sample sizes ranged from 37 to 200 participants per study. The mean age of participants across studies ranged from 21 to 37.7 years. The mean gestational age at intervention varied between 34.2 and 37.7 weeks. The dosing regimens for nifedipine included oral or sublingual administration, with initial doses typically ranging from 5 mg to 20 mg, followed by repeated doses every 15 to 30 min depending on blood pressure response. Hydralazine was administered intravenously, with initial doses ranging from 5 mg to 10 mg, followed by subsequent doses at similar intervals. The frequency and method of dose titration varied by study, with some using fixed intervals and others basing subsequent dosing on blood pressure measurements. The included trials are summarized in detail in Table 1. Five studies [2,9,10,13,14] were judged to have some concerns regarding risk of bias. These concerns were primarily related to issues in the randomization process (D1), deviations from intended interventions (D2), and missing outcome data (D3) (Figure S1). Only two studies [11,12] were assessed as having an overall low risk of bias across all domains.

### 3.3. Maternal Outcomes

#### 3.3.1. Time to Optimal BP Control

**Figure 2:**
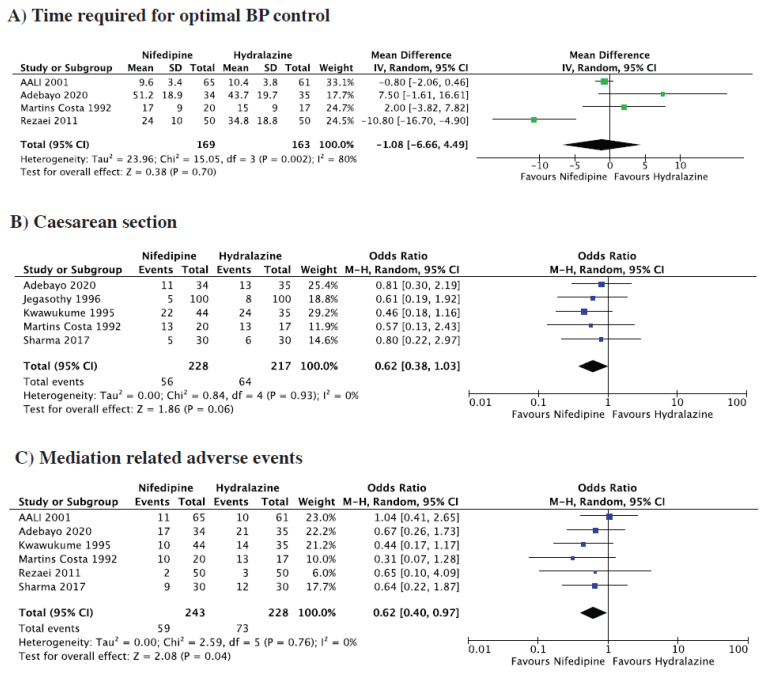
Forest plot for (**A**) Time required for optimal BP control, (**B**) Caesarean section, (**C**) Medication-related adverse events. Forest plot showing odds ratios (blue squares) and 95% confidence intervals (horizontal lines) for individual studies comparing Nifedipine and Hydralazine. The black diamond represents the overall pooled effect using a Mantel-Haenszel random-effects model, with its width indicating the 95% CI.

Four studies reported on time to optimal BP control. The pooled analysis demonstrated that comparable results between the two groups (MD = −1.08 min, 95% CI = −6.66 to 4.49; *p* = 0.70, I^2^ = 80%) (Figure 2A).

#### 3.3.2. Caesarean Section

Five studies reported about incidence of people caesarean section. The Pooled analysis showed that the odds are comparable between the two groups (OR = 0.62, 95% CI = 0.38 to 1.03; *p* = 0.06, I^2^ = 0%) (Figure 2B).

#### 3.3.3. Adverse Events

Five studies reported about medication related adverse events, which included headache, dizziness, nausea, vomiting, tacchycardia, and flushing. Pooled analysis indicated that patients treated with nifedipine had significantly lower odds of experiencing these adverse events compared to those receiving intravenous hydralazine (OR = 0.62, 95% CI = 0.40 to 0.97; *p* = 0.04, I^2^ = 0%) (Figure 2C).

### 3.4. Neonatal Outcomes

#### 3.4.1. Neonatal Birth Weight

**Figure 3:**
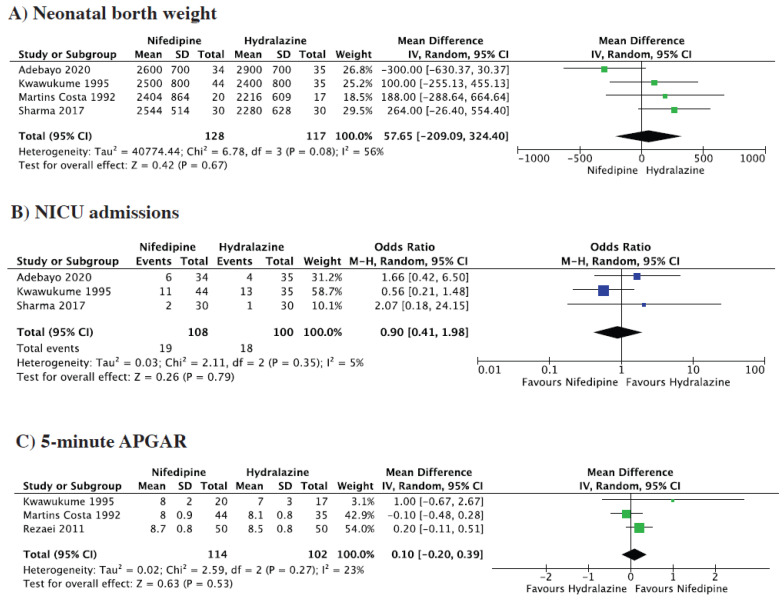
Forest plot for (**A**) Neonatal birth weight, (**B**) NICU admissions, (**C**) 5-min. Forest plot showing odds ratios (blue squares) and 95% confidence intervals (horizontal lines) for individual studies comparing Nifedipine and Hydralazine. The black diamond represents the overall pooled effect using a Mantel-Haenszel random-effects model, with its width indicating the 95% CI.

Neonatal birth weight stay was reported by three studies. The pooled analysis showed no statistically significant difference between the two groups (MD = 57.65 g, 95% CI = −209.09 to −324.40; *p* = 0.67, I^2^ = 56%) (Figure 3A).

#### 3.4.2. NICU Admission

Three studies reported the about NICU admissions, the pooled analysis demonstrated no significant difference between the two groups (OR = 0.90, 95% CI = 0.41 to 1.98; *p* = 0.79, I^2^ = 5%) (Figure 3B).

#### 3.4.3. 5-Min APGAR Score

Three studies reported on 5-min APGAR score. The pooled analysis demonstrated no significant difference between the two groups (MD = 0.1, 95% CI = −0.20 to 0.39; *p* = 0.53, I^2^ = 23%) (Figure 3C).

## 4. Discussion

This meta-analysis offers a comprehensive and up-to-date evaluation of the comparative efficacy of nifedipine and intravenous hydralazine in the management of severe hypertension during pregnancy. The findings indicate that both medications are similarly effective with respect to the time required to achieve optimal blood pressure control, rates of cesarean delivery, neonatal intensive care unit (NICU) admissions, neonatal birth weight, and APGAR scores. Notably, nifedipine demonstrated a more favorable safety profile, with significantly lower odds of adverse maternal effects compared to hydralazine.

Hypertensive disorders of pregnancy remain a leading cause of maternal and neonatal morbidity and mortality worldwide [15]. Among the pharmacologic options available, both oral or sublingual nifedipine and intravenous hydralazine have been widely used to manage severe hypertension during pregnancy [16]. Nifedipine, a calcium channel blocker, exerts its antihypertensive effect by reducing total peripheral resistance through vasodilation. In contrast, hydralazine acts as a direct arteriolar vasodilator. Multiple studies have assessed the efficacy and safety of these agents in controlling severe hypertension in pregnant women [16,17].

Our analysis confirms that both agents are effective in lowering blood pressure acutely, with no statistically significant difference in time to optimal blood pressure control and rates of cesarean delivery. This aligns with results prior studies which reported comparable onset of action between the two drugs [2,18]. However, unlike those prior reviews, our findings emphasize the significantly lower incidence of maternal adverse effects associated with nifedipine (95% CI: 0.40 to 0.97), a distinction that may bear important clinical implications, especially when treating patients at higher risk of drug-induced hypotension or tachycardia. However, nifedipine is relatively contraindicated in patients with significant aortic stenosis or left ventricular dysfunction due to its negative inotropic and vasodilatory effects [19]. In contrast, intravenous hydralazine may exacerbate symptoms in patients with systemic lupus erythematosus or lupus-like syndromes [20]. Nifedipine may be preferred in pregnant patients with asthma or autoimmune disorders, while hydralazine remains an option when oral administration is not feasible.

Hypertensive disorders of pregnancy are closely associated with an elevated risk of adverse neonatal outcomes, including low birth weight, prematurity, and the need for neonatal intensive care [21,22]. In our analysis, there were no significant differences between the nifedipine and hydralazine groups in terms of NICU admissions, neonatal birth weight, or 5-min APGAR scores. These findings suggest that both treatment options offer comparable safety profiles with respect to neonatal outcomes. This is consistent with previous literature, which showed no clear advantage of one agent over the other in terms of perinatal safety [23].

This evidence supports the use of oral nifedipine for the management of severe hypertension during pregnancy, particularly in settings where intravenous administration is less feasible. Its comparable efficacy, coupled with a more favorable maternal safety profile and potential neonatal benefits, make it a suitable alternative to intravenous hydralazine. Nevertheless, the choice of antihypertensive therapy should be individualized, taking into account the clinical scenario, drug availability, and patient-specific factors.

While this review provides a comprehensive comparison of nifedipine and hydralazine for the management of severe hypertension in pregnancy, several limitations must be acknowledged. First, there was notable heterogeneity among the included studies in terms of hypertension management protocols, drug administration, and dosing regimens. Rapid titration or higher doses of IV hydralazine have been associated with maternal hypotension and fetal distress due to abrupt blood pressure reductions, necessitating cautious dose escalation [24]. Similarly, higher or repeated doses of nifedipine, particularly immediate-release formulations, can lead to excessive vasodilation and maternal tachycardia, although it generally exhibits a more favorable safety profile [25]. Second, the meta-analysis relied on study-level data rather than individual participant data, which restricted our ability to conduct detailed subgroup analyses. As a result, we were unable to explore the potential influence of key baseline characteristics—such as gestational age at the time of treatment initiation, maternal comorbidities, or severity of hypertension—on treatment outcomes. Third, the relatively small number of included studies (fewer than ten) limited our ability to formally assess publication bias using funnel plot asymmetry or statistical tests such as Egger’s test. This raises the possibility that unpublished or selectively reported data could have influenced the findings. Lastly, the risk of bias assessment revealed some concerns in several key domains, particularly in the processes of randomization, deviations from intended interventions, and handling of missing outcome data. Inadequate reporting or execution in these areas may have introduced bias, potentially affecting the internal validity of the included studies.

## 5. Conclusions

Both nifedipine and intravenous hydralazine demonstrated comparable efficacy in achieving optimal blood pressure control and similar maternal and neonatal outcomes, including cesarean delivery rates, birth weight, NICU admissions, and 5-min APGAR scores. However, nifedipine was associated with a significantly lower risk of maternal adverse effects, suggesting better tolerability. These findings support the use of either agent in clinical practice, with a potential preference for nifedipine due to its more favorable safety profile.

## Figures and Tables

**Table 1:** Baseline characteristics of included studies.

Author (Year)	Sample Size		Age (Mean ± SD) *		Mean Gestational Age *		Drug Dose	
	Nifedipine	Hydralazine	Nifedipine	Hydralazine	Nifedipine	Hydralazine	Nifedipine	Hydralazine
Martins Costa 1992	20	17	21 ± 4	23 ± 6	36 ± 2	36 ± 2	10 mg PO (initial dose), 20 mg PO (subsequent doses) every 20 min up to 5 doses	5 mg IV (initial dose), 10 mg IV (subsequent doses) every 20 min up to 5 doses
Kwawukume 1995	49	49	34.3 ± 2.9	34.0 ± 3.4	34.3 ± 2.9	34.0 ± 3.4	10 mg PO (initial dose), 10–20 mg PO (subsequent doses) every 6–8 h until delivery.	5 mg IV (initial dose), 10 mg IV (subsequent doses) every 10–15 min, repeated based on blood pressure measurements.
Jegasothy 1996	100	100	28.2 ± 4.8	26.3 ± 4.2	35.3 ± 3.2	36.5 ± 2.9	5 mg sublingual (initial dose), 5 mg sublingual (subsequent doses) every 15 min up to 2 doses, then switch to Hydralazine infusion if DBP > 120 mmHg after 30 min.	5 mg IV (initial dose), 5 mg IV (subsequent doses) every 15 min up to 3 doses, then switch to Hydralazine infusion if DBP > 120 mmHg after 30 min.
AALI 2001	65	61	27.1 ± 6.4	26.8 ± 6.1	37 ± 3.3	37.7 ± 8.3	8 mg sublingual (initial dose), repeated every 20 min	5–10 mg IV (initial dose), repeated every 20 min as needed.
Rezaei 2011	25	25	29.4 ± 5.8	29.6 ± 6	35.6 ± 2.5	34.2 ± 3.3	10 mg PO (initial dose), 20 mg PO (subsequent dose) every 20 min up to 5 doses	5 mg IV (initial dose), 10 mg IV (subsequent doses) every 20 min up to 5 doses
Sharma 2017	30	30	23.4 ± 2.6	24.2 ± 6.3	36.7 ± 4.0	36.9 ± 2.4	10 mg PO every 20 min up to 4 doses	5 mg IV over 1 min, followed by 10 mg IV over 1 min every 20 min
Adebayo 2020	78	78	24.4 ± 4.3	24.6 ± 4.6	36.2 ± 6.4	37.0 ± 4.5	20 mg PO (initial dose), 20 mg PO (subsequent doses) every 30 min up to 5 doses.	10 mg IV (initial dose), 10 mg IV (subsequent doses) every 30 min up to 5 doses.

PO, oral route; IV, intravenous; DBO; diastolic blood pressure. * Continuous outcomes are reported as mean ± SD.

## Data Availability

All data generated or analyzed during this study are included in this article. Further inquiries can be directed to the corresponding author.

## Supplementary Materials

The following supporting information can be downloaded at: https://www.mdpi.com/article/10.3390/medsci13030091/s1, Figure S1: Risk of bias for RCTs; Table S1: Search Strategy.
