# Supplementary material for: Efficacy and Safety of Nifedipine Compared to Intravenous Hydralazine for Severe Hypertensive Disorders in Pregnancy: A Systematic Review and Meta-Analysis of Randmomized Controlled Trials
Source: Med Sci (Basel). 2025 Jul 13;13(3):91. doi: 10.3390/medsci13030091 (PMC12286209; doi:10.3390/medsci13030091)

Table S1: Search Strategy

Database	Search string	Records
PubMed/MEDLINE	("Nifedipine"[Mesh] OR nifedipine[tiab]) AND ("Hydralazine"[Mesh] OR hydralazine[tiab]) AND ("Hypertension, Pregnancy-Induced"[Mesh] OR "pre-eclampsia"[tiab] OR "severe hypertension"[tiab] OR "pregnancy-induced hypertension"[tiab]) AND ("Pregnancy"[Mesh] OR pregnant[tiab] OR pregnancy[tiab]) AND (randomized controlled trial[pt] OR controlled clinical trial[pt] OR randomized[tiab] OR placebo[tiab] OR "clinical trial"[tiab]) NOT (animals[mh] NOT humans[mh])	30
Cochrane	(nifedipine) AND (hydralazine) AND (pregnancy OR pregnant) AND ("severe hypertension" OR "pregnancy-induced hypertension" OR preeclampsia OR "pre-eclampsia")	30
Embase	('nifedipine'/exp OR nifedipine:ti,ab) AND ('hydralazine'/exp OR hydralazine:ti,ab) AND ('severe hypertension':ti,ab OR 'pregnancy-induced hypertension':ti,ab OR 'pre-eclampsia':ti,ab OR preeclampsia:ti,ab) AND ('pregnancy'/exp OR pregnancy:ti,ab OR pregnant:ti,ab) AND ([randomized controlled trial]/lim OR 'randomized controlled trial':ti,ab OR 'controlled clinical trial':ti,ab OR randomized:ti,ab OR placebo:ti,ab) NOT ([animals]/lim NOT [humans]/lim)	65

Figure S1: Risk of bias for RCTs

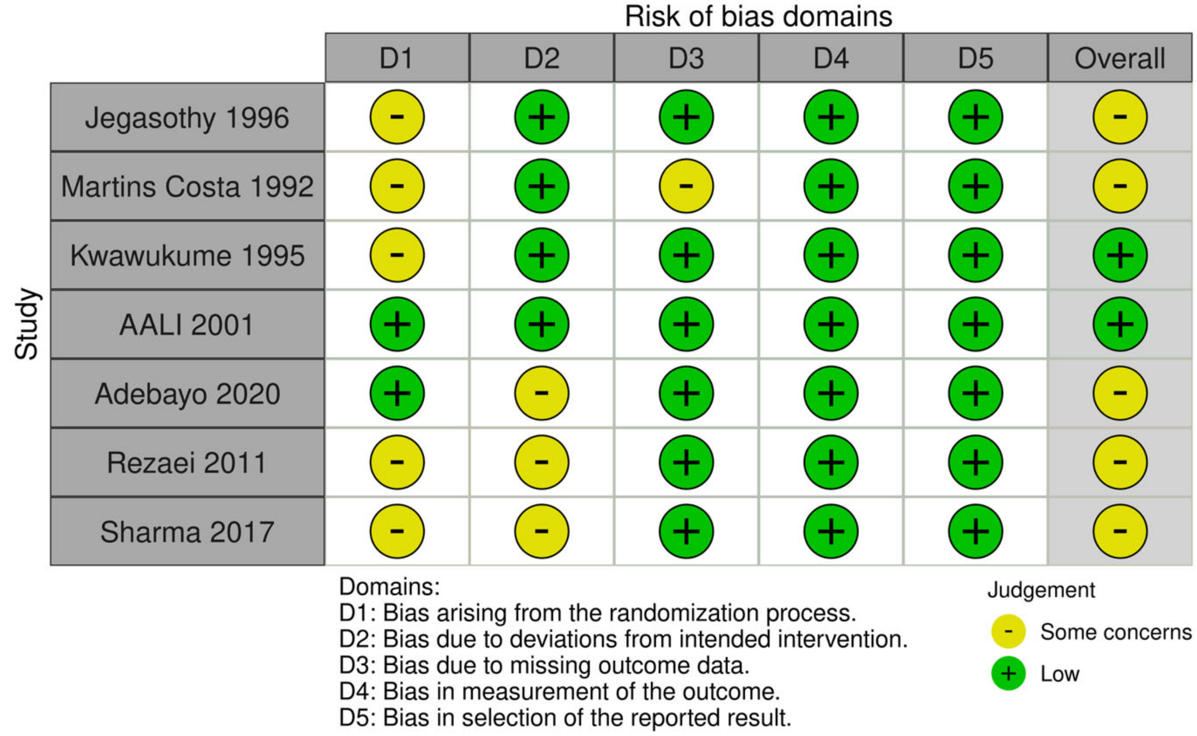

Supplement: Supplementary file 1 [file medsci-13-00091-s001.zip › medsci-3718178-supplementary.pdf]
